# Outcome stagnation of liver transplantation for primary sclerosing cholangitis in the Model for End-Stage Liver Disease era

**DOI:** 10.1007/s00423-014-1214-6

**Published:** 2014-06-04

**Authors:** Johannes Klose, Michelle A. Klose, Courtney Metz, Frank Lehner, Michael P. Manns, Juergen Klempnauer, Nils Hoppe, Harald Schrem, Alexander Kaltenborn

**Affiliations:** 1 General, Visceral and Transplant Surgery, University of Heidelberg, Im Neuenheimer Feld, 110, 69120 Heidelberg, Germany; 2 General, Visceral and Transplant Surgery, Hannover Medical School, Hannover, Germany; 3 Gastroenterology, Hepatology and Endocrinology, Hannover Medical School, Hannover, Germany; 4 CELLS—Centre for Ethics and Law in the Life Sciences, Leibniz University Hannover, Hannover, Germany; 5 Integrated Research and Treatment Center Transplantation (IFB-Tx), Hannover Medical School, Hannover, Germany; 6 Trauma and Orthopaedic Surgery, Federal Armed Forces Hospital, Westerstede, Germany

**Keywords:** MELD-based allocation, Cholangiocarcinoma, Outcome, Survival, Autoimmune liver disease, Multivariate analysis

## Abstract

**Purpose:**

Survival after liver transplantation (LTX) has decreased in Germany since the implementation of Model for end-stage liver disease (MELD)-based liver allocation. Primary sclerosing cholangitis (PSC) is known for its otherwise excellent outcome after LTX. The influence of MELD-based liver allocation and subsequent allocation policy alterations on the outcome of LTX for PSC is analyzed.

**Methods:**

This is a retrospective observational study including 126 consecutive patients treated with LTX for PSC between January 1, 1999 and August 31, 2012. The PSC cohort was further compared to all other indications for LTX in the study period (*n* = 1420) with a mean follow-up of 7.9 years (SD 3.2). Multivariate risk-adjusted analyses were performed. Alterations of allocation policy have been taken into account systematically.

**Results:**

Transplant recipients suffering from PSC are significantly younger (*p* < 0.001), can be discharged earlier (*p* = 0.018), and have lower 3-month mortality than patients with other indications (*p* = 0.044). The observed time on the waiting list is significantly longer for patients with PSC (*p* < 0.001), and there is a trend toward lower match MELD points in the PSC cohort (*p* = 0.052). No improvement in means of short-term mortality could be shown in relation to alterations of allocation policy within the MELD era (*p* = 0.375). Survival rates of the pre-MELD era did not differ significantly from those of the MELD era (*p* = 0.097) in multivariate risk-adjusted analysis. Patients in the MELD era suffered pre-transplant significantly more frequently from dominant bile duct stenosis (*p* = 0.071, *p* = 0.059, *p* = 0.048, respectively; chi^2^).

**Conclusions:**

Progress is stagnating in LTX for PSC. Current liver allocation for PSC patients should be reconsidered.

## Introduction

Primary sclerosing cholangitis (PSC) is a chronic inflammatory disease affecting both the small and large intrahepatic and extrahepatic bile ducts leading to biliary ectasis, strictures, and, consequently, cholestasis and liver cirrhosis [1]. Liver transplantation (LTX) represents the only curative treatment option for PSC. Patient’s survival after LTX reaches 82, 77, and 75 % at 1, 3, and 5 years, respectively [2, 3]. There is an increased risk of developing cholangiocarcinoma (CC) as well as the impact of concomitant inflammatory bowel disease (IBD) which appears to be present in 70–80 % of all PSC patients and an additionally increased risk of colorectal cancer [1, 4, 5].

Since the implementation of the Model for End-Stage Liver Disease (MELD) for donor liver allocation in Germany in January 2007, it has been suspected that patients with PSC may be disadvantaged by the sickest-first principle of MELD-based liver allocation. This notion is justified by the fact that PSC patients typically experience a comparatively stable disease over prolonged periods of time until they may decompensate and/or develop CC and thus may no longer be transplantable with reasonable outcome. MELD-based allocation is based on abnormal coagulation, impaired renal function, and high levels of serum bilirubin. In fact, only high bilirubin levels are constantly observed in PSC patients and were suggested to represent a significant predictive risk factor [6]. Therefore, conditions for the award of exception points were defined [7]. These conditions include ≥2 episodes of culture-proven bacteremia within the last 6 months or septic complications of cholangitis without a biliary tube or stent in situ and were introduced in active allocation on June 27, 2008 [8]. However, only a small proportion of PSC patients fulfill the qualifications for exception points, and the remaining patients are still at risk to develop CC while waiting years for LTX until they gain a sufficiently high MELD score due to increased liver failure. Therefore, allocation policies for patients suffering from PSC have been adapted subsequently on March 13, 2012 (e.g., assignment of 22 MELD points at day of listing) [9].

It can be assumed that current donor liver allocation for PSC patients may preclude optimal outcomes after LTX. The introduction of MELD-based liver allocation in Germany in December 2006 has decreased waiting list mortality from 20 to 10 % but, at the same time, has reduced posttransplant 1-year survival from almost 90 to below 80 % [10, 11]. Following MELD introduction, the regular allocation threshold has increased in Germany from a MELD of initially 25 to now 34 points. At the same time, the quality of donor organs has seen a continuous deterioration in most Eurotransplant countries over the last 10–15 years [11].

The current study investigates the outcome of LTX for PSC in the pre-MELD and MELD era, taking subsequent changes of allocation policy into account and systematically compares PSC patients with other indications for LTX. The PSC patient cohort is further analyzed in depth regarding differences between the pre-MELD and MELD era. It is intended to analyze whether the different allocation policies lead to an intended improvement of outcome or whether there was a stagnation of clinical scientific progress.

## Patients and methods

This is a retrospective observational analysis of 126 consecutive patients who received an LTX for PSC at Hannover Medical School between January 1, 1999 and August 31, 2012. A total of 1420 consecutive LTXs for other indications than PSC in the same time frame served as a control cohort for statistical comparison. Living donor transplantations were excluded because organ allocation was not based on the MELD score (*n* = 118; 7.6 %). The required minimum follow-up was 3 months. Mean follow-up was 7.9 years (SD 3.2). Patient’s data of the PSC cohort included age, sex, biochemical parameters (creatinine, bilirubin, international normalized ratio (INR), cholinesterase, (CHE), aspartate aminotransferase (AST), and alanine aminotransferase (ALT)) at date of listing and LTX and the absence or presence of IBD inclusive the onset of colorectal cancer. For those patients in the early years of this millennium where INR was not routinely determined, the value was estimated based on the prothrombin time. The laboratory MELD score (lab MELD) was calculated for all patients as described previously [12]. Additionally, the proportion of patients after implementation of the MELD score was classified into those who fulfilled the qualification for exception points and those who did not, according to policy changes on the June 27, 2008 and March 13, 2012 [8, 9, 12], resulting in the match MELD as described by Eurotransplant, which was used for actual organ allocation [12]. Furthermore, the influence of typical PSC complications (e.g., dominant bile duct stenosis and hepatobiliary (HB) surgery prior to transplantation or the onset of CC) on the outcome after LTX was analyzed. Donor characteristics like age, sex, graft type (split versus full organ), and cold ischemia time (CIT) were taken into account.

### Study endpoints

The primary study endpoints were 30-day mortality, 3-month mortality, and long-term survival after LTX. Secondary endpoints were the onset of surgical complications classified as proposed by Dindo et al. [13].

### Statistical analysis

Categorical variables were compared using the Pearson’s chi^2^ test (chi^2^); the Mann-Whitney *U* test (MWU) was used to compare continuous data. Means were compared with the Student’s two-sided *t* test. The study endpoints, 3-month mortality, 30-day mortality, and onset of complications were analyzed with binary logistic regression analysis. Survival analysis was performed with Kaplan-Meier estimation and Cox regression analysis. Relevant risk factors for survival were identified with univariate Cox regression. The alpha level in univariate Cox regression was set at *p* = 0.1 to ensure that also variables with comparatively small impact were considered for risk-adjusted analysis. Multivariate, risk-adjusted Cox proportional hazard models were then created including all significant variables from univariate analysis to identify relevant, independent risk factors for survival after LTX. For all other statistical tests, a *p* value <0.05 was defined as significant. Calculations were performed using SPSS 21.0 (IBM, Somers, NY, USA).

## Results

### Patient characteristics

Eighty-five (31 female = 36 %; 54 male = 64 %) of 959 patients (8.8 %) were transplanted for PSC in the pre-MELD era (January 1, 1999 to December 31, 2006); 41 (14 females = 34 %; 27 males = 66 %) of 487 patients (7.2 %) received a liver allograft due to PSC after the implementation of MELD-based allocation (*p* = 0.236; chi^2^). The percentage of pediatric patients (defined as <18 years) was significantly higher in the non-PSC cohort (<0.001; chi^2^). The PSC cohort had a median age of 40 years (range 3–66 years). Seventy-four of the 126 PSC patients (58.7 %) suffered from IBD prior to transplantation; 13 patients (10.3 %) were diagnosed with colon cancer prior to LTX. A split graft was transplanted in 20.6 % of all PSC cases (*n* = 26).

### Comparison of PSC patients with other indications leading to LTX in the MELD era

**Fig. 1:**
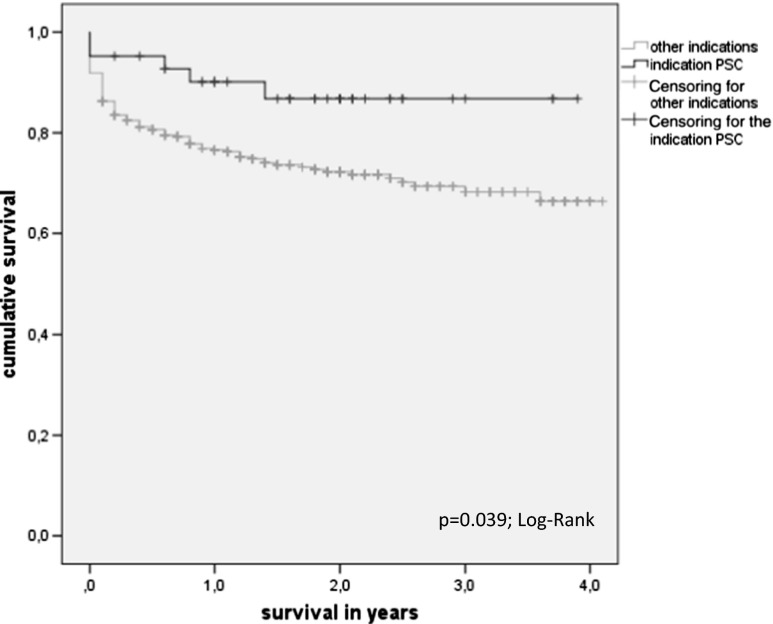
Survival of patients with PSC versus patients with other indications leading to LTX

Table 1 summarizes the results of the statistical comparison of patient characteristics and outcome data of transplant recipients with versus without PSC in the MELD era. Transplant recipients suffering from PSC are significantly younger (*p* < 0.001), can be discharged earlier (*p* = 0.018), and have lower 3-month mortality than patients with other indications (*p* = 0.044). The observed time on the waiting list is significantly longer for patients with PSC (*p* < 0.001), and there is a trend toward lower match MELD points in the PSC cohort, although this analysis does not reach statistical significance (*p* = 0.052). Recipients transplanted for PSC have significantly longer survival than all other indications for LTX (*p* = 0.039; log-rank) (see Fig. 1).

**Table 1:** The results of the comparison of patient characteristics and outcome data of transplant recipients with versus without PSC in the MELD era

	PSC cohort (*n* = 126)	Non-PSC cohort (*n* = 1420)	*p* value
Age at transplant (mean; SD)	43 (11)	50 (13)	<0.001^a^
Length of hospital stay (mean; SD)	35 (21)	53 (49)	0.018^a^
Operative duration (min) (mean; SD)	226 (51)	222 (71)	0.713^a^
CIT (min) (mean; SD)	616 (152)	573 (176)	0.095^a^
Survival (years) (mean; SD)	1.7 (0.9)	1.5 (1.2)	0.397^a^
Days on the waiting list (mean; SD)	854 (897)	294 (441)	<0.001^a^
Lab MELD (mean; SD)	19 (10)	21 (11)	0.387^a^
Match MELD (mean; SD)	22 (10)	26 (10)	0.052^a^
Necessity of postoperative revision	19 (45.2 %)	233 (57.7 %)	0.122^b^
3-month mortality	2 (4.9 %)	67 (16.9 %)	0.044^b^
30-day mortality	2 (4.9 %)	48 (11.8 %)	0.168^b^

*PSC* primary sclerosing cholangitis, *SD* standard deviation, *CIT* cold ischemic time

^a^Student’s *t* test

^b^Pearson’s chi^2^ test

### Patient survival

As summarized in Table 2, the multivariate Cox regression model for analysis of differing survival outcome in the pre-MELD versus the MELD era was risk-adjusted for CC prior to LTX, histologically proven CC in the explanted recipient liver, CC after LTX, first part of the MELD era (as defined above), and basiliximab as immunosuppressive induction therapy after LTX. All other investigated variables did not reach significance in univariate Cox regression analysis (*p* < 0.1). The multivariate Cox regression analysis revealed CC after LTX as the only independent risk factor for mortality (*p* < 0.001; HR 43.841; 95 % confidence interval (CI) 9.483–202.678). Neither being transplanted in the MELD era (*p* = 0.097) nor being one of the MELD sub-eras as defined by changes in allocation policy leads to an independently increased or decreased mortality risk in multivariate analysis (*p* = 0.055). Three-month mortality as well as 30-day mortality did not significantly differ between the pre-MELD and the MELD era (both *p* = 0.202, multivariate binary logistic regression analysis). No improvement in means of short-term mortality could be shown in relation to alterations of allocation policy within the MELD era (*p* = 0.375; multivariate binary logistic regression analysis).

**Table 2:** The results of the analysis of long-term survival of patients transplanted for PSC, which were significant in univariate and multivariate Cox regression analyses

	Univariate Cox regression		Multivariate Cox regression	
	*p* value	HR (95 % CI)	*p* value	HR (95 % CI)
MELD era yes/no	0.016	4.631 (1.357–15.803)	0.097	n.a.
CC prior to transplant	0.030	5.514 (1.481–20.526)	0.605	n.a.
CC in the recipient liver histology	<0.001	18.069 (5.300–61.601)	0.817	n.a.
CC after LTX	<0.001	43.841 (9.483–202.678)	<0.001	43.841 (9.483–202.678)
Part 1 MELD era	0.021	4.752 (1.260–17.928)	0.055	n.a.
Basiliximab induction	0.081	n.a.	0.345	n.a.

Part 1 MELD era: from introduction of MELD-based allocation (January 1, 2007) until first allocation change for PSC patients (June 27, 2008)

*MELD* Model for End-Stage Liver Disease, *HR* hazard ratio, *95 % CI* 95 % confidence interval, *CC* cholangiocarcinoma, *LTX* liver transplantation, *n.a.* not available

### Hepatic artery thrombosis and graft loss

Nine of 126 transplant recipients of the PSC cohort (7.4 %) developed a hepatic artery thrombosis (HAT) during follow-up, whereas HAT was observed in 59 non-PSC patients (4.5 %) in the same follow-up time (*p* = 0.120; chi^2^). Occurrence of HAT after LTX was a significant risk factor for graft loss (*p* < 0.001; HR 5.104; 95 % CI 3.867–6.738; Cox regression).

### Complications after LTX for PSC

The proportion of postoperative complications was comparable in both groups and displayed no significant differences between patients who were transplanted in the MELD era as compared to those patients transplanted in the pre-MELD era (*p* = 0.233; MWU; see Table 3). This result was confirmed by multivariate binary logistic regression analysis for the endpoint “postoperative complication yes/no,” which remained insignificant (*p* = 0.760).

**Table 3:** Surgical complications after LTX for PSC before and after implementation of MELD-based allocation (*p* = 0.233; MWU)

Clavien-Dindo score	Pre-MELD	MELD	Total
I	8	0	8
I–II	38	20	58
II	6	3	9
IIIa	5	2	7
IIIb	8	6	14
Iva	1	2	3
IVa	7	3	10
Ivb	1	1	2
IVb	9	2	11
V	2	1	3

Categorized with the Clavien-Dindo score

### Biochemical parameters and MELD scores

Biochemical parameters at the time of listing and at the day of LTX are summarized in Table 4. At the time of listing, patients in the MELD era have significantly higher lab MELD scores (*p* = 0.002), higher levels of bilirubin (*p* = 0.010), higher INR values (0.003), and higher AST levels (*p* = 0.008). At the day of LTX, significantly increased lab MELD scores (*p* < 0.001), higher bilirubin values (*p* < 0.001), increased INR levels (*p* = 0.006), as well as lower CHE levels (0.031) were observed in the MELD era.

**Table 4:** Patient characteristics and biochemical parameters at time of listing and at day of LTX

	Pre-MELD era (*n* = 85)	MELD era (*n* = 41)	*p* value
Age at the time of listing; median (range)	36 (2–62)	40 (15–61)	0.140^a^
Sex (females/males)	31 (36 %)/54 (64 %)	14 (34 %)/27 (66 %)	0.799^b^
Lab MELD at day of listing; mean (SD)	10 (4)	13 (7)	0.002^c^
Bilirubin (μmol/l) at day of listing; mean (SD)	50.7 (83.2)	107.2 (156.7)	0.010^c^
Creatinine (μmol/l) at day of listing; mean (SD)	61.5 (13.9)	65.9 (23.0)	0.262^c^
INR at day of listing; mean (SD)	1.1 (0.2)	1.2 (0.3)	0.003^c^
CHE (kU/l) at day of listing; mean (SD)	4.6 (2.1)	4.4 (2.0)	0.520^c^
AST (U/l) at day of listing; mean (SD)	62.1 (61.8)	98.6 (72.5)	0.008^c^
ALT (U/l) at day of listing; mean (SD)	76.9 (75.4)	94.4 (75.3)	0.231^c^
Lab MELD score at day of LTX; mean (SD)	11 (6)	18 (7)	<0.001^c^
Bilirubin (μmol/l) at day of LTX; mean (SD)	69.9 (119.6)	209.5 (234.2)	<0.001^c^
Creatinine (μmol/l) at day of LTX; mean (SD)	62.7 (24.1)	73.4 (36.3)	0.095^c^
INR at day of LTX; mean (SD)	1.2 (0.4)	1.4 (0.5)	0.006^c^
CHE (kU/l) at day of LTX; mean (SD)	4.7 (2.4)	3.7 (2.2)	0.031^c^
AST (U/l) at day of LTX; mean (SD)	112.9 (294.7)	117.3 (80.6)	0.900^c^
ALT (U/l) at day of LTX; mean (SD)	93.2 (101.2)	95.5 (67.8)	0.882^c^

^a^Mann-Whitney *U* test

^b^Pearson’s chi^2^ test

^c^Student’s *t* test

### Preoperative PSC-related features

Table 5 summarizes the clinical characteristics of investigated PSC patients. Patients in the MELD era suffered pre-transplantation more frequently from typical PSC-related complications, namely, recurrent cholangitis, dominant bile duct stenosis, and dysplasia in brush cytology. However, only the development of a dominant bile duct stenosis was significantly more frequent among patients in the MELD era (39 versus 23.5 %; *p* = 0.048; chi^2^). Six patients developed a re-manifestation of PSC during follow-up (4.8 %). Four percent of the PSC cohort (*n* = 5) were diagnosed with CC during follow-up. The presence of concomitant IBD was equally distributed between both groups. An active form or complicated course of the disease was more frequent among patients in the pre-MELD era without reaching statistical significance (*p* = 0.317).

**Table 5:** The frequencies of PSC-related features as well as preoperative and postoperative complications for all patients who underwent LTX for PSC

	Pre-MELD (*n* = 85)	MELD (*n* = 41)	*p* value (chi^2^)
Recurent cholangitis prior to LTX	20 (23.5 %)	16 (39 %)	0.071
Dominant bile duct stenosis	30 (35.3 %)	24 (58.5 %)	0.048
Dysplasia in brush cytology	5 (5.9 %)	7 (17.1 %)	0.059
CC prior to LTX	7 (8.2 %)	2 (4.9 %)	0.493
HB surgery prior to LTX	7 (8 %)	6 (14.6 %)	0.269
IBD prior to LTX	49 (57.6 %)	25 (61 %)	0.219
Active IBD prior to LTX	20 (40.8 %)	7 (28 %)	0.317
Colorectal cancer prior to LTX	9 (10.6 %)	4 (9.8 %)	0.954
Cirrhosis in explanted liver	60 (70.6 %)	30 (73.2 %)	0.522
CC in explanted liver	6 (7.1 %)	3 (7.3 %)	0.958
CC after LTX	5 (5.9 %)	1 (2.4 %)	0.395
Recurrent cholangitis after LTX	20(23.5 %)	7 (17.1 %)	0.408
Re-PSC after LTX	5 (6.4 %)	1 (2.6 %)	0.374
IBD relapse after LTX	3 (3.5 %)	0 (0 %)	0.223
Colorectal cancer after LTX	3 (3.5 %)	0 (0 %)	0.223
PTLD after LTX	1 (1.2 %)	0 (0 %)	0.486
Rejection after LTX	24 (28.2 %)	7 (17.1 %)	0.173

### Analysis of PSC patients in the MELD era

The mean time on the waiting list increased since introduction of MELD-based allocation from 1.6 to 2.3 years without reaching statistical significance (*p* = 0.068; *t* test). Twenty-six of 41 patients (63 %) in the MELD era fulfilled the criteria for exception points. Patients within the MELD era who gained exception points during their waiting time had a significantly longer overall waiting time (mean 2.67 (*n* = 26) versus 1.64 years (*n* = 15), *p* = 0.045; MWU). Nevertheless, waiting time from the first award of exception points until transplantation was significantly reduced to a mean waiting period of 191 days (median 110.5 days, range 0–487 days) as compared to cases without any exceptions points until LTX (mean 567 days, range 4–2,614 days) (*p* = 0.021, MWU). Cumulative survival among patients with or without exception points did not differ significantly (*p* = 0.634; log-rank).

### Postoperative PCS-related features

We further analyzed the course of PSC-associated co-morbidities after LTX. In the pre-MELD versus the MELD era, there was no statistical difference concerning the occurrence of CC, recurrent cholangitis, IBD relapse or active course of disease, the occurrence of colorectal cancer, the development of posttransplant lymphoproliferative disease (PTLD), or more than one treatable episode of rejection after LTX observed. Results are summarized in Table 5.

## Discussion

Against the background of currently diminishing donor numbers and a situation in the German transplant community which is further sharpened by several transplant scandals about manipulating liver transplant waiting lists, the current MELD-based donor liver allocation is widely discussed [14]. It is evident that the public expects trustworthy, transparent, and ethical donor organ allocation rules which enable optimal graft utilization and avoid donor organ waste in cases with foreseeable futile transplantation. After the introduction of MELD-based liver allocation, an increase in short-term mortality and sub-par survival rates after LTX has been observed [10, 15]. This observation casts serious doubts on the optimal utilization of donor livers with the current donor organ allocation policy. This in turn raises questions in relation to the ethical justifiability of the system as a whole and of a significant and indefensible systemic disadvantage to certain patient groups (e.g., PSC). Issues with distributive justice in MELD-based allocation systems have already been the subject of criticism [16]. Especially PSC patients, who have an otherwise excellent outcome as compared to other indications leading to LTX, could be disadvantaged by the current liver allocation policies [2, 17–20]. The survival rates for PSC patients after LTX are de facto excellent and exceed 75 % after 5 years in the USA and Scandinavia [2, 3, 21, 22]. The presented data supports this notion (see Fig. 1). Moreover, LTX is the only available curative therapy for PSC [18, 19].

It is undisputed that the short-term and long-term prognoses after LTX are largely dependent on the nature of the underlying liver disease, the overall morbidity and condition of the recipient, and donor liver quality, which is notoriously difficult to define [23–26]. This insight has lead to several so-called “standard exceptions” in MELD-based liver allocation, e.g., for cases with hepatocellular carcinoma or PSC [12]. Currently, about 60 % of all donor livers are allocated in Germany according to the original MELD score (so-called lab MELD) while the remainder are allocated due to standard exceptions, “non-standard exceptions,” and “rescue allocations” [11, 27]. In conclusion, this situation provided further motivation to analyze the current outcomes in LTX for PSC patients since the systematic introduction of MELD-based liver allocation in Germany.

Interestingly, patients who gained exception points during their waiting time had a longer overall waiting time for LTX. However, when analyzing the time from the first award of exception points until transplantation, mean waiting time is significantly shorter as compared to patients without any exception points during their waiting time. These findings confirm the assumption that listing with exception points reduces waiting time. We believe that posttransplant mortality and waiting time could be reduced, and finally, survival after LTX for PSC patients could be improved with the use of a more adequate donor liver allocation policy.

Our data indicates that the cumulative survival of PSC patients who underwent LTX has not declined significantly after the implementation of MELD-based allocation. This is an important difference compared to previously published data on outcomes after LTX for all indications in Germany in the pre-MELD versus the MELD era [10, 11, 15]. As expected, in the present cohort of PSC patients, mean lab MELD values were higher in the MELD era prior to LTX. This observation was previously shown for all indications for LTX in Germany [10]. This series shows, in line with previous publications, that higher lab MELD scores are associated with a deteriorated biochemical profile, including poor liver and kidney functions and poor coagulation [10]. After the introduction of MELD-based allocation in the present series, waiting time increased without reaching statistical significance. Patients who were transplanted in the MELD era had to wait longer (mean waiting time 2.3 years) for LTX as compared to previously presented data from Scandinavia and the USA (mean waiting time of 1 month and 1 year, respectively) [18, 21]. Noteworthy, in contrast to Germany and the USA, liver allocation in Scandinavia is not MELD-based [18].

The biggest concern for PSC patients is the development of disease-typical complications [1]. This includes primary dominant bile duct stenosis and, consequently, an increased risk for cholangitis, cholangiosepsis, and/or CC. Moreover, every dominant bile duct stenosis could mimic CC, whereas the differentiation between benign strictures and malignant stenosis might be difficult [28]. There are no distinct risk factors for the development of CC in PS; however, increased waiting time for LTX is assumed to increase the risk for CC. Although we observed a higher incidence of dominant bile duct stenosis accompanied with a trend toward higher frequency of cell dysplasia in biliary brush cytology among patients in the MELD era, there was no statistical difference in the incidence of CC before LTX or incidentally in the explanted livers as compared to the pre-MELD era. Thus, increased waiting time in the MELD era did not result in an increased incidence of CC (see Table 5). The reason for the higher frequency of dominant bile duct stenosis in the MELD era remains unclear. One speculation might be more thorough and possibly more frequent endoscopic examinations in the care of PSC patients within the last years.

Hepatobiliary surgery prior to LTX is known to affect the outcome negatively even after exclusion of patients with malignancy [3, 29, 30]. Although we observed a higher incidence within the MELD era, hepatobiliary surgery prior to LTX did not influence the outcome of LTX significantly in our cohort. Consequently, we cannot confirm that hepatobiliary surgery in PSC patients should be avoided due to a potential risk for poor survival after LTX as suggested before [3].

Concomitant IBD represents a relevant risk factor for reduced survival among PSC patients after LTX as well [30]. Neither the incidence of concomitant IBD nor the onset of colorectal cancer was represented differently in the pre-MELD versus the MELD era. However, in contrast to previous studies, we did not further differentiate the type of IBD [3, 30].

The rate of postoperative complications and re-transplants did not differ significantly between the pre-MELD and MELD era. It is well known that arterial thrombosis is not only the main indication for re-transplantation; it was also reported to occur more frequently in PSC patients [3, 23, 31]. In our cohort, we also identified arterial thrombosis as a primary reason leading to re-transplantation, followed by primary non-function.

PSC patients are known to suffer more frequently from one or more episodes of rejection after LTX as compared to patients who underwent LTX due to other liver diseases [32]. We observed in our cohort a higher frequency of rejections among patients in the pre-MELD era as compared to the MELD era without reaching statistical significance. The high frequency of rejections is in line with data from the literature reporting of 20–40 % of patients who will suffer from at least one episode of rejection [32, 33]. One or more episodes of rejection early after LTX are suspected to be associated with PSC recurrence [32]. Despite the long observation period, we only observed a low frequency of disease recurrence (6.4 %) compared to the data presented in previous studies (5.7–59.1 %) [32]. The difference in PSC recurrence before versus after implementation of MELD-based allocation did not reach statistical significance.

In a recently published analysis of the incidence and long-term risk of de novo malignancy in the liver transplant cohort from Hannover covering a total of 2,000 patients after LTX with a total of 14,490 person years of follow-up including 180 patients transplanted for PSC, we could not find a significantly increased risk for PTLD in these patients as compared to an age-matched normal population or as compared to other indications leading to LTX, but we did find an increased risk for colorectal carcinoma in PSC patients after transplantation [34]. It is therefore unsurprising that the incidence of PTLD after LTX was very low in this study.

One limitation of our single-center study is its retrospective character and potential center bias. Further, the number of patients is relatively small with a rather short follow-up time. Finally, we did not analyze patients who were removed from the waiting list due to severe complications such as advanced CC or death caused by liver failure. The influence of decreased donor organ quality over time in the present series is largely speculative, although an increased donor age after implementation of MELD-based allocation has been observed. However, it has been shown that current donor organ quality scores (DRI, ECD score, D-MELD score, ET-DRI) did not influence outcome after LTX in our center in the MELD era [24, 35].

Despite the fact that the criteria which must be fulfilled in order to gain exception points do not represent the whole spectrum of potential complications with prognostic relevance in PSC patients, the alterations of allocation policy during the study period seemed to be able to prevent further disadvantages of PSC patients in means of comparable outcomes with other indications leading to LTX. Nevertheless, only a deterioration of results could be prevented.

The data of this study underlines the conclusion that before and after the implementation of MELD-based allocation, there is no progress and rather stagnation in outcome after LTX for PSC, pointing to a lack of medical progress for these patients which is even more pronounced for patients with other indications for LTX who experience worse outcomes since the introduction of MELD-based allocation in Germany. These aspects warrant, at least in our view, the conclusion that liver allocation for PSC patients should be reconsidered. The delineation of what type of modifications would improve the long-term prognosis/outcome of PSC patients after transplantation needs to take into account the problem of ubiquitous donor organ shortage and the mechanisms of a justifiable distribution of this very rare resource, which needs to take into account all other indications for LTX and the fact that donation rates are currently significantly decreasing in Germany since 2012. The current study was not designed and able to provide a comprehensive solution to improve the current organ allocation dilemma that would have to take into account also further aspects such as urgency and utility of LTX. However, the current study does show the relevance of PSC-specific complications as well as the influence of recent alterations of allocation rules on outcome after transplantation for PSC. Surveillance of the influence of organ allocation rule changes on outcome for specific patient groups is necessary and provides valuable information for the ongoing and current debate on allocation rules.
